# Crystal structure of {(but-3-en-1-yl)bis­[(pyridin-2-yl)meth­yl]amine-κ^3^ *N*,*N*′,*N*′′}di­chlorido­copper(II) diethyl ether hemisolvate

**DOI:** 10.1107/S2056989015003448

**Published:** 2015-02-25

**Authors:** Katherine A. Bussey, Jennifer R. Connell, Annie R. McGlone, Margaret E. Mraz, Kayode D. Oshin, Tomislav Pintauer, Allen G. Oliver

**Affiliations:** a Department of Chemistry & Physics, Saint Mary’s College, Notre Dame, IN 46556, USA; b Department of Chemistry & Biochemistry, Duquesne University, Pittsburgh, PA 15282, USA; c Department of Chemistry & Biochemistry, University of Notre Dame, Notre Dame, IN 46556, USA

**Keywords:** crystal structure, five-coordinate copper(II) complex, Atom Transfer Radical Addition (ATRA) reactions

## Abstract

The five-coordinate Cu^II^ atom in the title complex [CuCl_2_(C_16_H_19_N_3_)]·0.5C_4_H_10_O, adopts a near-ideal square-pyramidal geometry (τ-5 = 0.01). The apical Cu—Cl distance is 0.2626 (6) Å longer than the basal Cu—Cl distance. Weak C—H⋯Cl interactions between pyridine rings and the Cl atoms of adjacent complex molecules are present. The solvent molecule, located on a twofold rotation axis, is situated in the voids of this arrangement. Copper atoms coordinated by tridentate nitro­gen-containing ligands have been found to be excellent promoters of Atom Transfer Radical Addition (ATRA) reactions.

## Abstract 2

The hemi-diethyl etherate of the square pyramidal copper complex, 1-butene-bis­(pyridin-2-ylmeth­yl)amine copper(II) chloride is reported. The basal plane consists of the three nitro­gen atoms from the ligand and one chlorine. The second chlorine occupies the apical position of the square pyramid.

## Chemical context   

Transition-metal-catalyzed Atom Transfer Radical Addition (ATRA) reactions of haloalkanes and α-halo­carbonyls to α-olefins have emerged as some of the most atom economical methods for simultaneously forming C—C and C—*X* bonds, leading to the production of more attractive mol­ecules with well-defined compositions, architectures, and functionalities (Pintauer & Matyjaszewski, 2005 ▸). Copper(I) complexes with tridentate and tetra­dentate nitro­gen-based ligands are currently some of the most active multidentate ligand structures developed for use in ATRA reactions (Matyjaszewski *et al.*, 2001 ▸). In view of the importance of these types of complexes, we report the synthesis and structural characterization of the title compound {(but-3-en-1-yl)bis­[(pyridin-2-yl)meth­yl]amine-κ^3^ *N*,*N*′,*N*′′} di­chlorido­copper(II) diethyl ether hemisolvate, (I). 
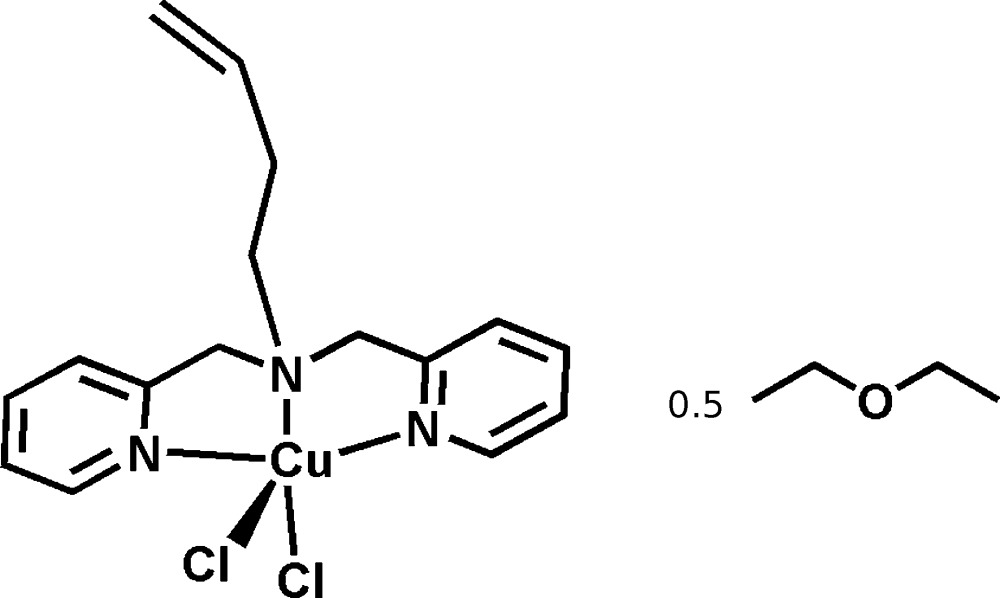


## Structural commentary   

**Figure 1:**
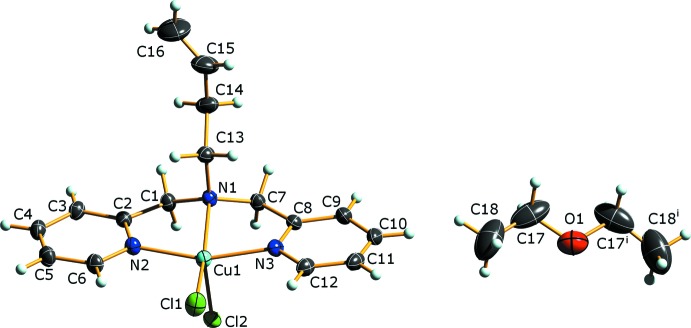
The molecular structure and atom-labeling scheme for (I). Displacement parameters are depicted at the 50% probability level. [Symmetry code: (i) −*x* + 1, *y*, −*z* + 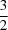.]

The title complex, (I) (Fig. 1 ▸), adopts a typical-for-this-class of compounds (*vide infra*), slightly distorted square-pyramidal geometry, as shown in the bond angles about the Cu^II^ atom. A τ-5 analysis of the distortions about the Cu^II^ atom yields a value of 0.01, close to an ideal value of zero for a perfect square-pyramidal geometry [Addison *et al.*, 1984 ▸; τ-5 = (β − α)/60 where β and α are the angles formed by atoms *trans* across the metal atom that do not include the apical atom]. In the complex, the Cu^II^ atom lies 0.2761 (5) Å out of the mean basal plane formed by the three coordinating N atoms and atom Cl1, reflecting the slight distortion from a true square plane. The Cu—N bond lengths are all similar [1.9980 (11)–2.0700 (10) Å] and the apical Cu—Cl2 distance is considerably longer [2.5134 (4) Å] than that of Cu—Cl1 [2.2508 (4) Å] in the basal plane. The diethyl ether mol­ecule of crystallization is located in the unit cell with the O atom on the crystallographic twofold rotation axis at [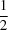, *y*, 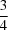].

## Supra­molecular features   

**Figure 2:**
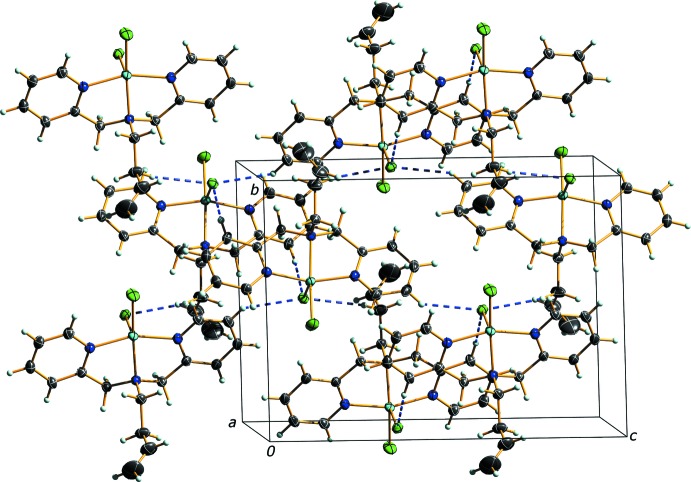
Packing diagram viewed along the *a* direction demonstrating the linear C—H⋯Cl electrostatic inter­actions (blue dashed lines).

Despite an open coordination site on the Cu^II^ atom, the complex does not dimerize through a chloride bridge, that is often observed in similar complexes (*vide infra*). There are weak electrostatic C—H⋯Cl inter­actions between pyridine rings and the basal chlorine of adjacent mol­ecules (Table 1 ▸ and Fig. 2 ▸). Close contacts about the butenyl chain are typical van der Waals contacts. The orientation of the butenyl chain is such that it is *anti* to the apical Cl ligand, effectively proximal to the vacant sixth coordination site of the Cu^II^ atom. Instead, the diethyl ether mol­ecule of crystallization is located in the pocket formed by the butenyl chain and the basal coordination plane of the Cu^II^ atom. Perhaps surprisingly, the ether O atom is not oriented towards, or spatially close to, the Cu atom [Cu⋯O1^ii^ = 4.9130 (9) Å; symmetry code (ii) −*x* + 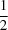, −*y* + 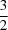, −*z* + 1] and merely serves to occupy a void space that would otherwise be formed by mol­ecular packing.

## Database survey   

Although there are 80 copper chloride structures that incorporate the bis­(pyridin-2-ylmeth­yl)amine ligand (Groom & Allen, 2014 ▸; CSD Version 5.36 plus one update), only 20 have a sole bis­(pyridin-2-ylmeth­yl)amine ligand chelating to a CuCl_2_ moiety within an overall five coordination. The remaining 60 structures either have a tethered pair or tethered tiplet of ligands, or have the bridging chlorines between two complexes and are thus the more common geometry adopted by copper coordinated by a bis­(pyridin-2-ylmeth­yl)amine based ligand. The geometry of the ligand and pendant group observed herein, is also a common feature of these structures, *vis-a-vis*, the pendant chain is oriented *anti* to the apical chlorine.

## Synthesis and crystallization   

For the preparation of (but-3-en-1-yl)bis­[(pyridin-2-yl)meth­yl]amine (see Scheme 1 below), the bis­(pyridin-2-ylmeth­yl)amine (BPMA) pre­cursor was synthesized and purified following literature procedures (Carvalho *et al.*, 2006 ▸). BPMA (8.064 g, 40.5 mmol) was dissolved in aceto­nitrile (15 ml) followed by the addition of tri­ethyl­amine (4.098 g, 40.5 mmol) and 4-bromo­butene (5.468 g, 40.5 mmol). The reaction vessel was sealed and allowed to mix for 4 d to ensure complete deprotonation and coupling occurred. Generation of the tri­ethyl­amine hydrogen bromide salt, Et_3_NH^+^·Br^−^, was observed as white crystals in the brown-colored solution. The mixture was filtered and the desired product extracted from the filtrate using a hexa­ne/water mixture. The hexane layer was separated and solvent removed to yield the ligand as a yellow colored oil (yield 8.516 g, 83%). The ligand was stored in a septum-sealed round-bottomed flask under argon gas in a refrigerator.
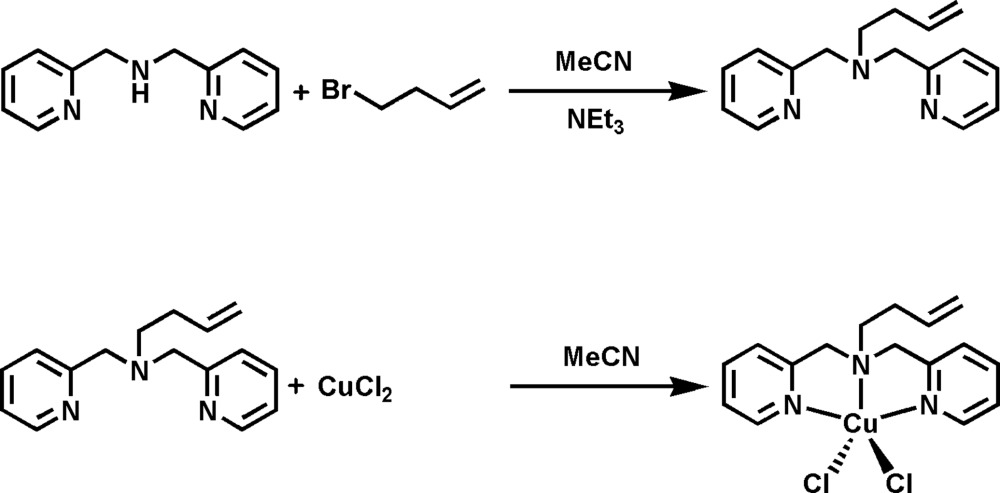


For the synthesis of the title compound, (I), 1-butene-BPMA (2.000 g, 7.900 mmol) was dissolved in aceto­nitrile (20 ml) in a 50 ml round-bottomed flask. CuCl_2_ (1.062 g, 7.900 mmol) was added to the flask to give a green-colored solution. The reaction was allowed to mix for 6 h, then pentane (20 ml) was added slowly to the solution to generate a bright-green precipitate. The solvent was removed from the round-bottomed flask by connecting it to a rotary evaporator. The precipitate obtained was washed twice by transferring two 15 ml aliquots of pentane into the flask and stirring vigorously for 30 min. The solvent was removed and the precipitate dried under vacuum for 2 h to yield a green solid (yield 2.909 g, 95%). Slow diffusion of diethyl ether into an aceto­nitrile solution of the complex at room temperature produced crystals of (I) suitable for X-ray analysis.

## Refinement details   

Crystal data, data collection and structure refinement details are summarized in Table 2 ▸. All non-H atoms were refined with anisotropic displacement parameters. H atoms were included in idealized positions, with C—H = 0.95 (aromatic), 0.98 (meth­yl), and 0.99 Å (ethyl­inic/methyl­ene). Methyl H atoms were allowed to rotate to minimize their electron-density contribution. The *U*_iso_(H) values were set at 1.5*U*_eq_(C) for methyl H atoms and at 1.2*U*_eq_(C) otherwise.

## Supplementary Material

Crystal structure: contains datablock(s) I, New_Global_Publ_Block. DOI: 10.1107/S2056989015003448/pk2546sup1.cif

Structure factors: contains datablock(s) I. DOI: 10.1107/S2056989015003448/pk2546Isup2.hkl

CCDC reference: 1050406

Additional supporting information:  crystallographic information; 3D view; checkCIF report

## Figures and Tables

**Table 1:** Hydrogen-bond geometry (, )

*D*H*A*	*D*H	H*A*	*D* *A*	*D*H*A*
C3H3Cl2^i^	0.95	2.67	3.5541(15)	156
C11H11Cl2^ii^	0.95	2.74	3.4767(15)	135
C14H14*A*Cl2^iii^	0.99	2.80	3.7127(18)	153

Symmetry codes: (i) 
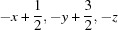
; (ii) 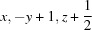; (iii) 
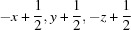
.

**Table 2:** Experimental details

Crystal data	
Chemical formula	[CuCl_2_(C_16_H_19_N_3_)]0.5C_4_H_10_O
*M*_r_	424.84
Crystal system, space group	Monoclinic, *C*2/*c*
Temperature (K)	150
*a*, *b*, *c* ()	22.1614(13), 11.5738(5), 16.4530(7)
()	108.771(1)
*V* (^3^)	3995.6(3)
*Z*	8
Radiation type	Mo *K*
(mm^1^)	1.37
Crystal size (mm)	0.50 0.28 0.10

Data collection	
Diffractometer	Bruker APEXII
Absorption correction	Multi-scan (*SADABS*; Krause *et al.*, 2015 ▸)
*T*_min_, *T*_max_	0.471, 0.840
No. of measured, independent and observed [*I* > 2(*I*)] reflections	24823, 6872, 5660
*R*_int_	0.025
(sin /)_max_ (^1^)	0.758

Refinement	
*R*[*F* ^2^ > 2(*F* ^2^)], *wR*(*F* ^2^), *S*	0.028, 0.072, 1.03
No. of reflections	6872
No. of parameters	223
H-atom treatment	H-atom parameters constrained
_max_, _min_ (e ^3^)	0.53, 0.43

Computer programs: *APEX2* and *SAINT* (Bruker, 2010 ▸), *SHELXS97* (Sheldrick, 2008 ▸), *SHELXL2014* (Sheldrick, 2015 ▸), *DIAMOND* (Brandenburg, 2006 ▸), *POV-RAY* (Cason, 2013 ▸) and *publCIF* (Westrip, 2010 ▸).

## supplementary crystallographic information

### Crystal data

**Table:**

[CuCl_2_(C_16_H_19_N_3_)]·0.5C_4_H_10_O	*F*(000) = 1760
*M**_r_* = 424.84	*D*_x_ = 1.412 Mg m^−^^3^
Monoclinic, *C*2/*c*	Mo *K*α radiation, λ = 0.71073 Å
*a* = 22.1614 (13) Å	Cell parameters from 8841 reflections
*b* = 11.5738 (5) Å	θ = 2.2–32.2°
*c* = 16.4530 (7) Å	µ = 1.37 mm^−^^1^
β = 108.771 (1)°	*T* = 150 K
*V* = 3995.6 (3) Å^3^	Rhomboid, blue
*Z* = 8	0.50 × 0.28 × 0.10 mm

### Data collection

**Table:**

Bruker APEXII diffractometer	6872 independent reflections
Radiation source: fine-focus sealed tube	5660 reflections with *I* > 2σ(*I*)
Graphite monochromator	*R*_int_ = 0.025
Detector resolution: 8.33 pixels mm^-1^	θ_max_ = 32.6°, θ_min_ = 1.9°
φ and ω scans	*h* = −32→33
Absorption correction: multi-scan (*SADABS*; Krause *et al.*, 2015)	*k* = −17→17
*T*_min_ = 0.471, *T*_max_ = 0.840	*l* = −23→24
24823 measured reflections	

### Refinement

**Table:**

Refinement on *F*^2^	Primary atom site location: structure-invariant direct methods
Least-squares matrix: full	Secondary atom site location: difference Fourier map
*R*[*F*^2^ > 2σ(*F*^2^)] = 0.028	Hydrogen site location: inferred from neighbouring sites
*wR*(*F*^2^) = 0.072	H-atom parameters constrained
*S* = 1.03	*w* = 1/[σ^2^(*F*_o_^2^) + (0.0348*P*)^2^ + 2.0749*P*] where *P* = (*F*_o_^2^ + 2*F*_c_^2^)/3
6872 reflections	(Δ/σ)_max_ = 0.002
223 parameters	Δρ_max_ = 0.53 e Å^−^^3^
0 restraints	Δρ_min_ = −0.43 e Å^−^^3^

### Special details

**Table:**

Geometry. All e.s.d.'s (except the e.s.d. in the dihedral angle between two l.s. planes) are estimated using the full covariance matrix. The cell e.s.d.'s are taken into account individually in the estimation of e.s.d.'s in distances, angles and torsion angles; correlations between e.s.d.'s in cell parameters are only used when they are defined by crystal symmetry. An approximate (isotropic) treatment of cell e.s.d.'s is used for estimating e.s.d.'s involving l.s. planes.

### Fractional atomic coordinates and isotropic or equivalent isotropic displacement parameters (Å^2^)

**Table:**

	*x*	*y*	*z*	*U*_iso_*/*U*_eq_	
Cu1	0.15616 (2)	0.59899 (2)	0.13662 (2)	0.01989 (5)	
Cl1	0.09795 (2)	0.43892 (3)	0.13435 (2)	0.03418 (8)	
Cl2	0.25976 (2)	0.52731 (3)	0.12375 (2)	0.02394 (7)	
N1	0.18137 (5)	0.77181 (9)	0.14348 (6)	0.01978 (19)	
N2	0.11788 (5)	0.64014 (9)	0.01190 (7)	0.0215 (2)	
N3	0.19179 (5)	0.61566 (9)	0.26406 (7)	0.0223 (2)	
C1	0.19332 (6)	0.79568 (11)	0.06162 (8)	0.0219 (2)	
H1A	0.2363	0.7679	0.0650	0.026*	
H1B	0.1915	0.8800	0.0508	0.026*	
C2	0.14371 (6)	0.73526 (11)	−0.01065 (8)	0.0205 (2)	
C3	0.12747 (6)	0.77160 (12)	−0.09536 (8)	0.0240 (2)	
H3	0.1460	0.8393	−0.1099	0.029*	
C4	0.08364 (6)	0.70725 (13)	−0.15849 (8)	0.0275 (3)	
H4	0.0728	0.7289	−0.2171	0.033*	
C5	0.05600 (7)	0.61125 (13)	−0.13490 (9)	0.0287 (3)	
H5	0.0251	0.5674	−0.1770	0.034*	
C6	0.07399 (6)	0.57991 (12)	−0.04920 (9)	0.0263 (3)	
H6	0.0549	0.5141	−0.0331	0.032*	
C7	0.23897 (6)	0.78269 (11)	0.22018 (8)	0.0223 (2)	
H7A	0.2440	0.8638	0.2403	0.027*	
H7B	0.2773	0.7607	0.2053	0.027*	
C8	0.23224 (6)	0.70480 (11)	0.29021 (8)	0.0219 (2)	
C9	0.26692 (7)	0.72191 (12)	0.37617 (9)	0.0284 (3)	
H9	0.2956	0.7850	0.3935	0.034*	
C10	0.25854 (8)	0.64431 (13)	0.43618 (9)	0.0315 (3)	
H10	0.2825	0.6525	0.4952	0.038*	
C11	0.21516 (7)	0.55514 (13)	0.40935 (9)	0.0298 (3)	
H11	0.2076	0.5034	0.4499	0.036*	
C12	0.18308 (7)	0.54244 (12)	0.32283 (9)	0.0263 (3)	
H12	0.1540	0.4802	0.3042	0.032*	
C13	0.12613 (6)	0.83899 (11)	0.15197 (9)	0.0250 (2)	
H13A	0.1182	0.8139	0.2052	0.030*	
H13B	0.0879	0.8187	0.1031	0.030*	
C14	0.13334 (8)	0.97031 (13)	0.15463 (11)	0.0370 (3)	
H14A	0.1719	0.9922	0.2024	0.044*	
H14B	0.1387	0.9976	0.1003	0.044*	
C15	0.07651 (10)	1.02672 (16)	0.16678 (14)	0.0517 (5)	
H15	0.0648	1.0031	0.2150	0.062*	
C16	0.04196 (14)	1.1043 (2)	0.1180 (2)	0.0866 (9)	
H16A	0.0519	1.1306	0.0691	0.104*	
H16B	0.0064	1.1356	0.1308	0.104*	
O1	0.5000	0.71720 (17)	0.7500	0.0552 (5)	
C17	0.46509 (12)	0.7851 (2)	0.6791 (2)	0.0809 (9)	
H17A	0.4948	0.8292	0.6570	0.097*	
H17B	0.4381	0.8409	0.6973	0.097*	
C18	0.42468 (16)	0.7095 (4)	0.6110 (2)	0.1069 (12)	
H18A	0.4016	0.7564	0.5610	0.160*	
H18B	0.3941	0.6687	0.6323	0.160*	
H18C	0.4515	0.6531	0.5942	0.160*	

### Atomic displacement parameters (Å^2^)

**Table:**

	*U*^11^	*U*^22^	*U*^33^	*U*^12^	*U*^13^	*U*^23^
Cu1	0.02130 (8)	0.01992 (8)	0.01888 (8)	−0.00387 (5)	0.00706 (6)	0.00069 (5)
Cl1	0.03288 (17)	0.03282 (17)	0.03443 (18)	−0.01494 (14)	0.00746 (14)	0.00510 (13)
Cl2	0.02487 (14)	0.02391 (14)	0.02458 (14)	−0.00119 (11)	0.01009 (11)	−0.00181 (11)
N1	0.0212 (5)	0.0208 (5)	0.0182 (5)	−0.0017 (4)	0.0076 (4)	−0.0003 (4)
N2	0.0209 (5)	0.0229 (5)	0.0205 (5)	−0.0029 (4)	0.0064 (4)	−0.0002 (4)
N3	0.0255 (5)	0.0233 (5)	0.0203 (5)	0.0010 (4)	0.0106 (4)	0.0013 (4)
C1	0.0241 (6)	0.0234 (6)	0.0193 (5)	−0.0051 (4)	0.0083 (5)	−0.0001 (4)
C2	0.0202 (5)	0.0217 (6)	0.0207 (5)	−0.0004 (4)	0.0081 (4)	−0.0004 (4)
C3	0.0223 (6)	0.0292 (6)	0.0214 (6)	0.0015 (5)	0.0082 (5)	0.0034 (5)
C4	0.0254 (6)	0.0367 (7)	0.0195 (6)	0.0040 (5)	0.0058 (5)	0.0017 (5)
C5	0.0243 (6)	0.0344 (7)	0.0237 (6)	−0.0024 (5)	0.0024 (5)	−0.0041 (5)
C6	0.0228 (6)	0.0290 (7)	0.0250 (6)	−0.0049 (5)	0.0048 (5)	−0.0016 (5)
C7	0.0247 (6)	0.0214 (6)	0.0199 (5)	−0.0034 (4)	0.0061 (5)	−0.0011 (4)
C8	0.0251 (6)	0.0212 (6)	0.0202 (5)	0.0029 (4)	0.0081 (5)	−0.0011 (4)
C9	0.0364 (7)	0.0254 (6)	0.0211 (6)	0.0046 (5)	0.0060 (5)	−0.0027 (5)
C10	0.0433 (8)	0.0305 (7)	0.0196 (6)	0.0122 (6)	0.0089 (6)	0.0005 (5)
C11	0.0383 (7)	0.0302 (7)	0.0251 (6)	0.0110 (6)	0.0161 (6)	0.0078 (5)
C12	0.0303 (6)	0.0267 (6)	0.0257 (6)	0.0031 (5)	0.0142 (5)	0.0056 (5)
C13	0.0271 (6)	0.0240 (6)	0.0255 (6)	0.0033 (5)	0.0107 (5)	0.0002 (5)
C14	0.0349 (8)	0.0245 (7)	0.0458 (9)	0.0048 (6)	0.0048 (7)	−0.0025 (6)
C15	0.0609 (12)	0.0382 (9)	0.0612 (12)	0.0139 (8)	0.0269 (10)	−0.0075 (8)
C16	0.0783 (18)	0.0776 (18)	0.095 (2)	0.0474 (15)	0.0156 (16)	−0.0047 (15)
O1	0.0521 (11)	0.0461 (11)	0.0708 (13)	0.000	0.0244 (10)	0.000
C17	0.0600 (14)	0.0728 (17)	0.124 (2)	0.0280 (13)	0.0496 (16)	0.0421 (16)
C18	0.0760 (19)	0.158 (4)	0.078 (2)	0.036 (2)	0.0130 (16)	0.035 (2)

### Geometric parameters (Å, º)

**Table:**

Cu1—N3	1.9980 (11)	C8—C9	1.3895 (18)
Cu1—N2	2.0093 (11)	C9—C10	1.391 (2)
Cu1—N1	2.0700 (10)	C9—H9	0.9500
Cu1—Cl1	2.2508 (4)	C10—C11	1.383 (2)
Cu1—Cl2	2.5134 (4)	C10—H10	0.9500
N1—C1	1.4800 (15)	C11—C12	1.379 (2)
N1—C7	1.4837 (16)	C11—H11	0.9500
N1—C13	1.4942 (16)	C12—H12	0.9500
N2—C6	1.3466 (16)	C13—C14	1.527 (2)
N2—C2	1.3469 (16)	C13—H13A	0.9900
N3—C8	1.3434 (17)	C13—H13B	0.9900
N3—C12	1.3455 (16)	C14—C15	1.488 (2)
C1—C2	1.5067 (17)	C14—H14A	0.9900
C1—H1A	0.9900	C14—H14B	0.9900
C1—H1B	0.9900	C15—C16	1.281 (3)
C2—C3	1.3878 (17)	C15—H15	0.9500
C3—C4	1.3883 (19)	C16—H16A	0.9500
C3—H3	0.9500	C16—H16B	0.9500
C4—C5	1.383 (2)	O1—C17^i^	1.413 (3)
C4—H4	0.9500	O1—C17	1.413 (3)
C5—C6	1.3845 (19)	C17—C18	1.476 (4)
C5—H5	0.9500	C17—H17A	0.9900
C6—H6	0.9500	C17—H17B	0.9900
C7—C8	1.5077 (17)	C18—H18A	0.9800
C7—H7A	0.9900	C18—H18B	0.9800
C7—H7B	0.9900	C18—H18C	0.9800
			
N3—Cu1—N2	160.62 (5)	H7A—C7—H7B	108.3
N3—Cu1—N1	80.84 (4)	N3—C8—C9	121.93 (12)
N2—Cu1—N1	81.05 (4)	N3—C8—C7	115.75 (11)
N3—Cu1—Cl1	97.34 (3)	C9—C8—C7	122.30 (12)
N2—Cu1—Cl1	97.28 (3)	C8—C9—C10	118.31 (13)
N1—Cu1—Cl1	159.94 (3)	C8—C9—H9	120.8
N3—Cu1—Cl2	93.27 (3)	C10—C9—H9	120.8
N2—Cu1—Cl2	95.11 (3)	C11—C10—C9	119.57 (13)
N1—Cu1—Cl2	94.92 (3)	C11—C10—H10	120.2
Cl1—Cu1—Cl2	105.136 (14)	C9—C10—H10	120.2
C1—N1—C7	113.65 (9)	C12—C11—C10	118.92 (13)
C1—N1—C13	112.33 (10)	C12—C11—H11	120.5
C7—N1—C13	112.52 (10)	C10—C11—H11	120.5
C1—N1—Cu1	104.77 (7)	N3—C12—C11	121.94 (13)
C7—N1—Cu1	105.76 (7)	N3—C12—H12	119.0
C13—N1—Cu1	107.05 (8)	C11—C12—H12	119.0
C6—N2—C2	119.11 (11)	N1—C13—C14	116.05 (11)
C6—N2—Cu1	127.29 (9)	N1—C13—H13A	108.3
C2—N2—Cu1	113.44 (8)	C14—C13—H13A	108.3
C8—N3—C12	119.27 (11)	N1—C13—H13B	108.3
C8—N3—Cu1	114.07 (8)	C14—C13—H13B	108.3
C12—N3—Cu1	126.42 (9)	H13A—C13—H13B	107.4
N1—C1—C2	109.43 (10)	C15—C14—C13	110.80 (14)
N1—C1—H1A	109.8	C15—C14—H14A	109.5
C2—C1—H1A	109.8	C13—C14—H14A	109.5
N1—C1—H1B	109.8	C15—C14—H14B	109.5
C2—C1—H1B	109.8	C13—C14—H14B	109.5
H1A—C1—H1B	108.2	H14A—C14—H14B	108.1
N2—C2—C3	121.90 (12)	C16—C15—C14	125.8 (2)
N2—C2—C1	115.39 (10)	C16—C15—H15	117.1
C3—C2—C1	122.65 (11)	C14—C15—H15	117.1
C2—C3—C4	118.80 (12)	C15—C16—H16A	120.0
C2—C3—H3	120.6	C15—C16—H16B	120.0
C4—C3—H3	120.6	H16A—C16—H16B	120.0
C5—C4—C3	119.17 (12)	C17^i^—O1—C17	112.4 (3)
C5—C4—H4	120.4	O1—C17—C18	109.5 (2)
C3—C4—H4	120.4	O1—C17—H17A	109.8
C4—C5—C6	119.20 (13)	C18—C17—H17A	109.8
C4—C5—H5	120.4	O1—C17—H17B	109.8
C6—C5—H5	120.4	C18—C17—H17B	109.8
N2—C6—C5	121.79 (13)	H17A—C17—H17B	108.2
N2—C6—H6	119.1	C17—C18—H18A	109.5
C5—C6—H6	119.1	C17—C18—H18B	109.5
N1—C7—C8	109.24 (10)	H18A—C18—H18B	109.5
N1—C7—H7A	109.8	C17—C18—H18C	109.5
C8—C7—H7A	109.8	H18A—C18—H18C	109.5
N1—C7—H7B	109.8	H18B—C18—H18C	109.5
C8—C7—H7B	109.8		
			
C7—N1—C1—C2	155.62 (10)	C12—N3—C8—C9	1.93 (19)
C13—N1—C1—C2	−75.20 (13)	Cu1—N3—C8—C9	−172.73 (10)
Cu1—N1—C1—C2	40.65 (11)	C12—N3—C8—C7	−179.82 (11)
C6—N2—C2—C3	−1.47 (18)	Cu1—N3—C8—C7	5.52 (14)
Cu1—N2—C2—C3	174.32 (10)	N1—C7—C8—N3	22.88 (15)
C6—N2—C2—C1	−178.80 (11)	N1—C7—C8—C9	−158.88 (12)
Cu1—N2—C2—C1	−3.00 (13)	N3—C8—C9—C10	−0.6 (2)
N1—C1—C2—N2	−26.50 (15)	C7—C8—C9—C10	−178.72 (12)
N1—C1—C2—C3	156.20 (11)	C8—C9—C10—C11	−1.8 (2)
N2—C2—C3—C4	−0.47 (19)	C9—C10—C11—C12	2.7 (2)
C1—C2—C3—C4	176.66 (12)	C8—N3—C12—C11	−0.91 (19)
C2—C3—C4—C5	2.0 (2)	Cu1—N3—C12—C11	173.03 (10)
C3—C4—C5—C6	−1.7 (2)	C10—C11—C12—N3	−1.4 (2)
C2—N2—C6—C5	1.8 (2)	C1—N1—C13—C14	−63.24 (15)
Cu1—N2—C6—C5	−173.32 (10)	C7—N1—C13—C14	66.53 (14)
C4—C5—C6—N2	−0.2 (2)	Cu1—N1—C13—C14	−177.71 (10)
C1—N1—C7—C8	−152.29 (10)	N1—C13—C14—C15	−177.66 (13)
C13—N1—C7—C8	78.63 (12)	C13—C14—C15—C16	−125.1 (3)
Cu1—N1—C7—C8	−37.91 (11)	C17^i^—O1—C17—C18	−174.1 (3)

Symmetry code: (i) −*x*+1, *y*, −*z*+3/2.

### Hydrogen-bond geometry (Å, º)

**Table:**

*D*—H···*A*	*D*—H	H···*A*	*D*···*A*	*D*—H···*A*
C3—H3···Cl2^ii^	0.95	2.67	3.5541 (15)	156
C11—H11···Cl2^iii^	0.95	2.74	3.4767 (15)	135
C14—H14*A*···Cl2^iv^	0.99	2.80	3.7127 (18)	153

Symmetry codes: (ii) −*x*+1/2, −*y*+3/2, −*z*; (iii) *x*, −*y*+1, *z*+1/2; (iv) −*x*+1/2, *y*+1/2, −*z*+1/2.
